# Overlap between substance and behavioural addictions: substance abuse in patients with pathological gambling

**DOI:** 10.1192/j.eurpsy.2022.2155

**Published:** 2022-09-01

**Authors:** N. Cunha E Costa, S. Cruz, G. Sobreira

**Affiliations:** Hospital Garcia de Orta, Almada, Psychiatry, Almada, Portugal

**Keywords:** Addiction, gambling, substance abuse, impulse-control disorders

## Abstract

**Introduction:**

Pathological gambling consists of a persistent and maladaptive pattern of gambling behavior, that often leads to significant adverse psychosocial and financial outcomes. It is currently classified as an “Impulse Disorder” on ICD-10 but the DSM-5 moved this diagnosis from “Impulse-Control Disorders” to “Substance-Related and Addictive Disorders” section^[1]^. Behavioral addictions, especially pathological gambling, share many features with substance dependences, namely clinical findings and behavioural patterns, comorbidity with psychiatric disorders, genetic factors and family history, neurobiology, natural history and response to treatment^[2]^.

**Objectives:**

To study the impact of substance abuse in patients with pathological gambling.

**Methods:**

Literary review, using PubMed database search, regarding substance abuse and pathological gambling.

**Results:**

57,5% of individuals with pathological gambling also present with some form of substance use^[3]^.There was also a large percentage of patients presenting with nicotine dependence (60,1%) and a fourfold increase in the risk of developing an alcohol use disorder^[3]^. Individuals with substance use disorders also show a threefold risk of developing pathological gambling and substance use appears to negatively influence gambling behaviours in this population. Gambling habits in adolescents have been linked to an increased risk of current and lifetime drug use of multiple substances^[4]^. Other psychiatric comorbidities were also frequent in this population: 37.9% of patients presented with mood disorders and 37.4% with anxiety disorders^[3]^.

**Conclusions:**

There is a significant clinical and neurobiological overlap between substance use disorders and pathological gambling. Individuals with pathological gambling have a high prevalence of substance use disorders and an increased lifetime risk of substance use, which negatively influences gambling behavior.

**Disclosure:**

No significant relationships.

